# Somatic Mutation Profiling of Intrahepatic Cholangiocarcinoma: Comparison between Primary and Metastasis Tumor Tissues

**DOI:** 10.1155/2020/5675020

**Published:** 2020-09-17

**Authors:** Shi-Feng Xu, Yuan Guo, Xin Zhang, Xiao-Dan Zhu, Ning Fan, Zhi-Lei Zhang, Gui-Bing Ren, Wei Rao, Yun-Jin Zang

**Affiliations:** ^1^Shandong Provincial Hospital Affiliated to Shandong First Medical University, Shandong, China; ^2^Liver Disease Center, The Affiliated Hospital of Qingdao University, Qingdao, China; ^3^Origimed, Shanghai, China; ^4^Organ Transplant Center, The Affiliated Hospital of Qingdao University, Qingdao, China; ^5^Department of Hepatobiliary Surgery, Fourth Hospital of Hebei Medical University, Shijiazhuang, China; ^6^Oncology Department, Armed Police Characteristic Medical Center, Tianjin, China; ^7^Division of Hepatology, Liver Disease Center, Organ Transplantation Center, The Affiliated Hospital of Qingdao University, Qingdao, China

## Abstract

**Introduction:**

Intrahepatic cholangiocarcinoma (ICC) exhibited increasing incidence and mortality around the world, with a 35% five-year survival rate. In this study, the genetic alteration of primary ICC and metastasis ICC was exhibited to discover novel personalized treatment strategies to improve the clinical prognosis.

**Methods:**

Based on 153 primary and 49 metastasis formalin-fixed paraffin-embedded ICC samples, comprehensive genomic profiling was carried out.

**Results:**

In primary tumor samples (PSs) and metastasis tumor samples (MSs), the top alteration genes were TP53 (41.8% vs 36.7%), KRAS (30.7% vs 36.7%), and ARID1A (22.2% vs 14.2%). In the top 20 most frequent alteration genes, BRAF showed lower mutation frequency in MSs as compared to PSs (0 vs 11.1%, *P*=0.015), while LRP1B exhibited opposed trend (22.4% vs 10.4%, *P*=0.032). In PSs, patients with MSI-H showed all PDL1 negative, and patients with PDL1 positive exhibited MSS both in PSs and MSs. It was found that the Notch pathway had more alteration genes in MSI-H patients (*P*=0.027). Furthermore, the patients with mutated immune genes in PSs were more than that in MSs (28.8% vs 8.2%, *P*=0.003, odd ratio = 0.2). Interestingly, the platinum drug resistance pathway was only enriched by mutated genes of MSs.

**Conclusions:**

In this study, the identification of two meaningful mutated genes, BRAF and LRP1B, highly mutated immune gene harbored by primary ICC patients. Both in PSs and MSs, no patients with MSI-H showed PDL1 positive. The Notch pathway had more alteration genes in patients with MSI-H. And the enrichment of the platinum drug resistance pathway in MSs might offer reference for the novel therapeutic strategy of ICC.

## 1. Introduction

Liver cancer, the fourth common causes of cancer death, had brought about 1.76 million deaths worldwide reported by the World Health Organization (WHO, https://www.who.int/news-room/fact-sheets/detail/cancer) in 2018. As one of the most frequent type of primary liver cancers, intrahepatic cholangiocarcinoma (ICC) exhibited increasing incidence and mortality around the world [1, 2]. For patients who suffered from early or resectable ICC, the possible method of cure is hepatectomy [3]. However, even after surgery, the clinical outcomes of patients are still not too optimistic, with about a 30%–35% five-year survival rate [2, 3], which was around 25% higher than that which have unresetable disease [4]. Hence, the improvement of clinical prognosis is urgent issues for ICC patients.

There are various kinds of factors contributing to low survival rate. One of them is the high incidence of recurrence of ICC [5], so patients with metastasis ICC need an effective and specific curative method to better survival rate. With more and more applications of next generation sequencing (NGS) technology, its advantages in diagnosis and treatment of solid and hematologic cancers also put growing attention [6, 7]. As well known, patients with cancer comprise mass of genomic alterations, which not only consists of driver alteration resulting in selective growth advantage to cancer cell but also passenger alteration. And NGS can facilitate to discriminate them to archive targetable therapy [8]. Besides, NGS also confers novel molecular biomarkers, such as tumor mutation burden (TMB) and microsatellite instability (MSI), which enhance the precision of clinical decisions [9]. For example, Chae et al. found that genetic alterations in DNA damage repair genes were observably related with longer progression-free survival of patients suffering from biliary tract cancer [10]; Chen et al. discovered that cholangiocarcinoma patients with dMMR status and a high level of TMB may have a same therapeutic effect with anti-PD-1-directed treatment [11]. Recently, somatic mutation landscape of ICC was reported, which might discover promising candidate driver alterations for cure of ICC [12]. Given that the high recurrence rate of ICC is an important reason for its low survival rate, the genetic landscape of primary and secondary tumor tissues was exhibited, so that more reference for precision of clinical decisions could be provided. That was not revealed before.

In our study, 202 of ICC patient samples, including 153 primary tumors and 49 metastasis tumors, performed NGS. The genetic alteration, TMB, MSI, and PDL1 expression were all measured or counted to reveal the difference between primary and metastasis ICC tumors. We hope that it can be offered more reference for personalized diagnosis to extend overall survival of ICC patients.

## 2. Materials and Methods

### 2.1. Patients and Samples

**Figure 1:**
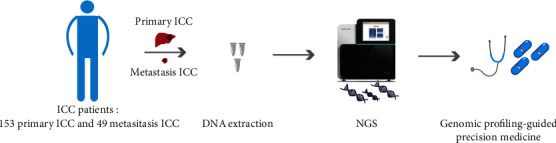
The workflow.

202 ICC tumor specimens, containing 153 primary tumor tissues and 49 metastasis tissues, were collected in this study. Each specimen had blood sample being regarded as a reference to detect somatic alterations. Clinical characteristics of all patients are demonstrated in Table 1. And the workflow is shown in Figure 1.

### 2.2. DNA Extraction

Before the extraction process, 4 *μ*m section of stained formalin-fixed paraffin-embedded (FFPE) sample was examined by the pathologist, so that each FFPE sample had the area of 1 cm^2^ or more and 20% tumor cellularity. From 10 of 4 *μ*m FFPE samples, 0.5∼2 *μ*g of DNA were generated. In the meantime, 200 *μ*L of whole blood from the paired FFPE samples was used to extract 1∼5 *μ*g DNA as normal control.

### 2.3. Library Construction and Hybridization Capture

A total of 50∼250 ng double-stranded DNA was interrupted ultrasonically to∼250 bp. The following library construction process was conducted using the KAPA Hyper Prep Kit according to the manual.

A custom hybrid capture panel encompassing more than 23,660 individually synthesized 5′-biotinylated DNA 120 bp oligonucleotides to target approximately 2.6 Mb of human genome, which contains 7029 exons of 468 cancer-related genes and selected introns of 39 genes that are often rearranged in cancer. Hybridization capture was in the light of the protocol of “Hybridization capture of DNA libraries using xGen® Lockdown® Probes and Reagents” (Integrated DNA Technologies, Version 3) and sequenced on an Illumina Nextseq 500 with mean coverage∼1000x. According to the protocol, comprehensive genomic profiling was carried out via the Yuansu assay (OrigiMed, China) paired end sequencing (2 × 75 bp). To estimate sequencing error rate, a PhiX spike-in was treated as an external control, counting the proportion of reads with 0–4 mismatches on the basis of the method described before [13].

### 2.4. Bioinformatics Pipeline for Single Nucleotide Variation (SNV) and Short Indels, Long Indels, Copy Number Alternations (CNA), and Gene Rearrangement

The raw reads were aligned to the human genome reference sequence (hg19) using the Burrows–Wheeler Aligner (BWA, v 0.6.2). Subsequently, PCR duplicates were removed by MarkDuplicates algorithm from Picard (version 1.47, http://picard.sourceforge.net/). And the details of bioinformatics pipeline for single nucleotide variation (SNV) and short indels, long indels, copy number alternations (CNA), and gene rearrangement had been reported by Jingyu Cao et al. [14].

### 2.5. The Examination of TMB, MSI, and PDL1

TMB score, MSI status, and PDL1 expression of each sample were calculated or assessed as previously described [14].

### 2.6. Statistical Analysis

Comparison between two groups was carried out with the Fisher test, Wilcoxon test, and chi-square test in *R* studio. *P* value < 0.05 was considered significant.

## 3. Result

### 3.1. Patient Characters

Patients involved in presented study comprised by 153 primary ICCs and 49 secondary ICCs. The primary ICCs consisted of 53 females and 100 males, with age ranging from 18 to 79, median value 60, while secondary ICC included 14 females and 35 males, with age ranging from 24 to 83, median value 59. The distribution of patients in both age and gender showed no statistical difference between PSs and MSs (Table 1).

### 3.2. Landscape of Somatic Mutations of PSs and MSs

**Figure 2:**
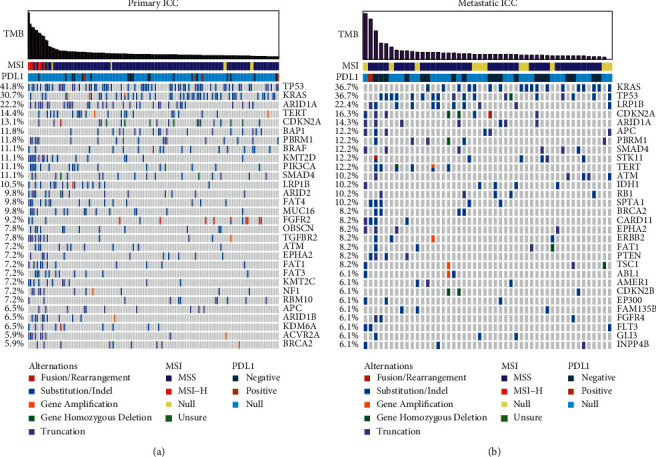
Mutational landscape of primary intrahepatic cholangiocarcinoma (ICC) (a) and metastatic ICC (b).

To explore the difference of gene alteration between PSs and MSs, the landscape of somatic mutations of PSs and MSs were exhibited. In primary tumor samples (PSs), the top 5 genes with the highest mutation frequency were TP53 (41.8%), KRAS (30.7%), ARID1A (22.2%), TERT (14.3%), and CDKN2A (13.1%), while in metastasis tumor samples (MSs), they similarly were TP53 (36.7%), KRAS (36.7%), LRP1B (22.4%), CDKN2A (16.3%), and ARID1A (14.2%, Figures 2(a) and 2(b)). In PSs, there were more alteration genes (468 genes) with 1777 variants, whereas in MSs, 484 variants happened in 243 genes. Of all genetic alterations, 40.2% (909/2261) was the variant of uncertain (or unknown) significance, which consisted by 40.6% (722/1777) in PSs and 38.6% (187/484), which indicated that the investigation on genetic alterations inducing function changing was still needed. Given that human chromosome 13 had been implied, involved in the development of part of liver cancers [15], the mutation frequency of gene located on it was compared. It was found that, in chromosome 13, the alterations in MSs were remarkable more than that in PSs (5.2% vs 2.7%, *P*=0.018). The variation type of PSs and MSs did not show significant difference in SNV, CNV, fusion, and long indel (83.7% vs 82.9%, 12.0% vs 13.3%, 2.4% vs 1.6%, and 1.98% vs 2.2%). The number of patients carrying any gene alteration in 11 pathways, such as DDR, PI3K, and WNT pathways (30.7% vs 30.6%, 30.1% vs 38.8%, and 22.2% vs 22.4%), also exhibited no remarkable difference compared to patients with no mutation (Figure S1).

### 3.3. MSI, PDL1, and TMB

**Figure 3:**
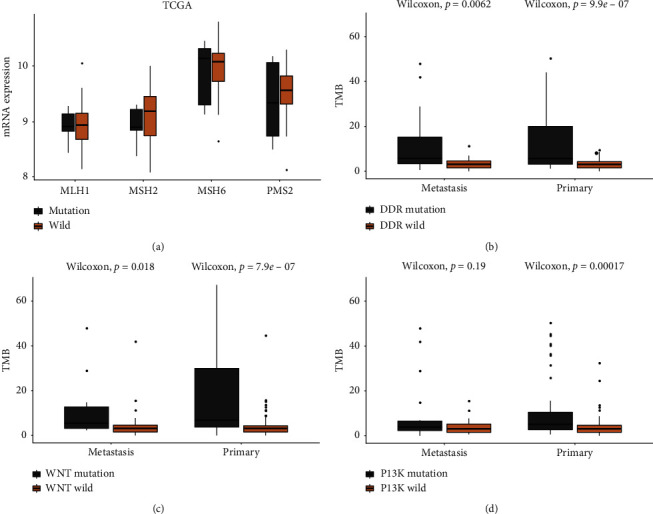
MSI and TMB. (a) The MSI-related gene expression from TCGA in the Notch pathway mutation and wild patient is shown. (B–D) The association of the TMB value and DDR pathway mutation (b), WNT pathway mutation (c), or PI3K pathway mutation (d) in primary ICC and metastatic ICC.

According to the NCCN guidelines, pembrolizumab (anti-PD1) was recommended for advanced cholangiocarcinoma with MSI-H [16, 17]. In this study, the MSI and PDL1 expression of ICC was also examined. Of 202 ICC patients, 184 had MSI status report, including 146 PSs and 38 MSs. Of 146 PSs, 7 was MSI-H, while in 38 MSs, no sample presented MSI-H. Most of patients showed MSS both in PSs (95.5%, 139/146) and MSs (100%, 38/38). In 23 PSs that had PDL1 measurement, 4 of them were positive, with positive score TPS 10%, TPS 20%, TPS 20%, and TPS 90%, respectively, while in 21 MSs, only 1 patient exhibited positive, with TPS 3%. A majority of ICC patients had PDL1 negative not only in PSs (82.6%, 19/23) but also in MSs (95.2%, 20/21). And in 44 of patients with both MSI and PDL1 detection, 1 of PSs with MSI-H showed PDL1 negative, while 5 of ICC patients (4 PSs and 1 MSs) with PDL1 positive exhibited MSS. To explore the MSI-H-related pathway, the alteration gene in patients with MSI-H was screened out and annotated with pathway. It was found that the Notch pathway had more alteration genes in MSI-H patients (*P*=0.027). The mRNA expression of MLH1, MSH2, MSH6, and PMS2, which was used to indicate MSI status from 36 cholangiocarcinoma patients, was downloaded from TCGA. In the light of whether patients harbored with the Notch pathway gene alteration or not, 36 cholangiocarcinoma patients were divided into the mutation group and wild group. The expression of MSH2 in the mutation group was lower than that in the wild group (Figure 3(a)).

To explore the relationship between TMB and pathway in PSs and MSs, all patients were grouped to the pathway mutation group and pathway wild group by whether harboring pathway gene mutation or not. It was found that the TMB distribution of PSs and MSs in the DDR mutation group and DDR wild group had a remarkable difference (*P* = 9.9*e *− 07 vs 0.0062, Figure 3(b)). In the WNT mutation group and WNT wild group, the TMB distribution of PSs and MSs demonstrated similar trend (*P* = 7.9*e*−07 vs 0.0018, Figure 3(c)), whereas in the PI3K mutation group and PI3K wild group, that of MSs showed no difference (*P* = 0.00017 vs 0.19, Figure 3(d)).

### 3.4. Genes with Differentially Mutation Frequency in PSs and MSs

**Figure 4:**
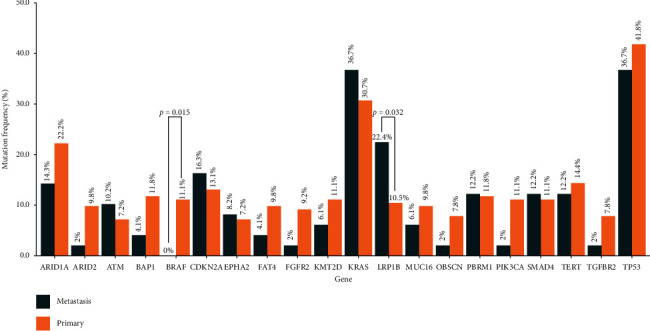
Mutational frequencies of the top 20 genes in primary ICC and metastatic ICC.

In the top 20 most frequently alteration genes, BRAF showed lower mutation frequency in MSs as compared to PSs (0% vs 11.1%, *P*=0.015), while LRP1B exhibited opposed trend (22.4% vs 10.4%, *P*=0.032, Figure 4).

After comparing the mutation frequency of each gene, 9 genes (BRAF, TSC1, LRP1B, EPCAM, GNA13, MYCL, PARP2, YES1, and STK24) exhibited statistically significant difference with *P* value < 0.05 (Table 2). Among them, BRAF and MYCL were confirmed as oncogene; LRP1B and TSC1 were tumor suppressor genes (TSG). BRAF, TSC1, and PARP2 were major factors of the RAS pathway, PI3K pathway, and DDR pathway. Besides, the variation type of nine genes was also observed and annotated on OncoKB (https://www.oncokb.org/). However, only BRAF alterations had more comprehensive annotated results, which showed that most of the mutations made BRAF gain-of-functions (Table 3), such as V600 E and D594 G. And V600 E had been reported as a drug target in various cancer types [18–22].

### 3.5. Mutated Immune Genes and Enrichment Analysis

**Figure 5:**
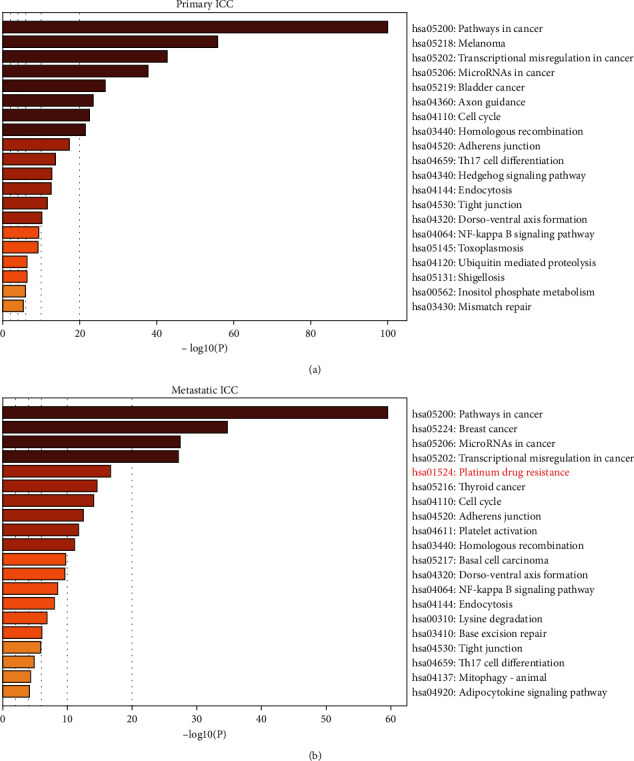
KEGG pathway and GO annotation analysis.

According to whether carried with mutated immune genes or not, patients were group into mutation and wild cohort. The patients with mutated immune genes in PSs were more than that in MSs (28.8% vs 8.2%, *P*=0.003, odd ratio = 0.2). In addition, the type of immunity cells affected by mutated genes in PSs was more multitudinous than that in MSs (Table 4). In order to investigate the pathway and GO function of mutation genes in PSs and MSs, the enrichment analysis was performed on Metascape (http://metascape.org/gp/index.html). Most of the enriched pathways or GO function was similar, but some diverse pathways or functions were still revealed. For example, platinum drug resistance was only enriched by mutated genes of MSs (Figures 5(a) and 5(b)).

## 4. Discussion

ICC patients present unoptimistic clinical outcomes mostly for the reason of the high relapse rate and low unresetable proportion, which indicates the urgency of emerging of new treatment strategy. In our study, the genetic alteration landscape of PSs and MSs was compared to unveil the potential personalized therapeutic options for ICCs. In this research, the mutation frequency of TP53 and KRAS of PSs was 41.8% (64/153) and 30.7% (47/153) and that of MSs was both 36.7% (18/49). The frequency of TP53 was similar to previous reports, but the frequency of KRAS was higher than earlier reports [12].

Of importance, in the top frequently mutated genes, we uncovered two genes, LRP1B and BRAF, with a significant difference between PSs and MSs (Figure 4). LRP1B, a member of the low-density lipoprotein receptor family and an important tumor suppressor gene, had been reported giving raise to increasing mutation burden in melanoma, pointing to high-TMB [23]. The semblable findings were also discovered in lung cancer and melanomas [23–26]. In present study, in all ICCs, encompassing PSs and MSs, the LRP1B mutation-type group had higher TMB, compared to the LRP1B wild-type group (median value: 7.0 vs 3.1; *P* = 5.532*e* − 07), which was in line with previous report. Of 49 MSs, 22.4% (11/49) harbored LRP1B mutation, which was higher than PSs (10.4%, 16/153; *P*=0.031). But, the TMB in PSs and MSs did not exhibit same trend, with median value as 3.7 vs 3.1 (*P*=0.76). The other gene, BRAF, encoding a protein attributing to the RAF family of serine/threonine protein kinases, takes part in tumor cell growth, invasion, and early diagnosis in melanoma [27, 28], and BRAF mutation was connected with metastatic disease in consensus molecular subtype-1 (CMS1) MSS cancers, resulting in poor prognosis in primary colorectal cancer [29]. However, in our study, BRAF mutation seemed not to show associated with metastasis. In PSs, 11% (17/153) harbored with BRAF mutation, while in MSs, no patient was discovered carrying it (*P* = 0.015). The results above might imply that the BRAF-mutant clone could not expand to metastasis region, although this mutation contributed to cell proliferation, which consisted with the findings in glioma [30]. The overwhelming majority of mutation of BRAF was conducive to gain-of-function and plays a role of oncogene, with high frequency of V600 E variant, which had been uncovered as a treatment target in papillary thyroid cancer, lung cancer, and colorectal cancer [31–33]. Besides, it was found that the alteration frequency of genes from chromosome 13 in MSs was significantly higher than that in PSs (5.2% vs 2.7%, *P*=0.018). The kind of mutated genes in PSs and MSs was similar, such as tumor suppressor gene BRCA2 and RB1, but the alteration frequency had typically declined in PSs, which might suggest that genes in chromosome 13 play an important role in tumor metastasis as reported in prostate cancer [34]. The remaining genes with remarkable different alteration frequency showed low mutational frequency both in PSs and MSs. Therefore, their description was not involved in the discussion.

Subsequently, it was unveiled that in MSs, whether PI3K pathway mutated or not, had no association with TMB value distribution (*P*=0.19). However, in PSs, the PI3K pathway mutation group demonstrated higher TMB, compared to the PI3K pathway wild group (*P*=0.00017). TMB of the DDR pathway mutation group and the WNT pathway mutation group was both higher than that of the DDR pathway wild group and the WNT pathway wild group, no matter in MSs or PSs (Figures 3(b)‐3(d)). These results might indicate that the PI3K pathway mutation in MSs did not affect TMB value in MSs with ICC, although the PI3K pathway had been uncovered promoting metastasis in various kinds of cancers [35–37]. Furthermore, except the null result of MSs, the remaining MSs showed MSS, and only one ICC metastasis patient expressed PDL1. In PSs, patients with MSI-H showed PDL1 negative. Patients with PDL1 positive exhibited MSS. Given that MSI-H patients responded better to anti-PD1 therapy [38, 39], the result of it might explain that immunotherapy had very weak efficacy.

The mutation of immune gene of PSs and MSs was observed. The result showed that 28.8% (44/153) PSs harbored immune gene mutation, while in MSs, only 8.2% (4/49) patients harbored it (*P*=0.003, odds ratio = 0.2). In addition, the type of immunity cells affected by mutated genes in PSs was more multitudinous than that in MSs (Table 4). We supposed the reason might be the type of immunity cells affected by mutated genes in MSs involved in vital segment of tumor cell metastasis or tumor cell caused clonal evolution after leaving from the primary site. Mast cells and dendritic cells were both discovered presenting promoting metastasis of tumor cells in nonsmall cell lung cancer cells [40] and metastatic melanoma [41], which supported the assumption above. The decline of the variety of genome alteration in MSs was also according with the “founder effect” theory, compared to PSs [30]. Another interesting finding was that in MSs, the platinum drug resistance pathway was enriched by the mutated genes (Figure 5). Anamaria Brozovic and Yuan Qin et al. uncovered that epithelial–mesenchymal transition (EMT) was associated with platinum drug resistance in ovarian cells and hepatocellular carcinoma [42, 43]. Although the phenomena that platinum exposure could result in obtaining EMT-like phenotype or EMT-derived metastasis was not unveiled in ICC [44, 45], the finding presented in our study also partly confirmed it. And we reckoned that it should be put more concentration on to improve the clinical outcomes of ICC.

## 5. Conclusions

In conclusion, our investigation exhibited the mutation landscape of primary ICC and metastasis ICC and found two meaningful mutated genes, BRAF and LRP1B, which had significantly different alteration frequency between MSs and PSs. Both in PSs and MSs, no patients with MSI-H showed PDL1 positive. The Notch pathway had more alteration genes in patients with MSI-H. Furthermore, it was found that the number of primary ICC patients harbored with immune gene was more than that of metastasis ICC patients. In MSs, mutated genes were enriched in the platinum drug resistance pathway, which was not presented in PSs.

## Figures and Tables

**Table 1:** Clinical characteristic of patients.

	Primary ICC cohort (*N* = 153)	Secondary ICC cohort (*N* = 49)	*P* value
Age (years): median (range)	60 (18–79)	59 (24–83)	0.54
Gender			
Female	53	14	0.54
Male	100	35	

**Table 2:** Genes with differentially mutation frequency in PSs and MSs.

Gene	Alteration frequency in primary ICC (%)	Alteration frequency in metastatic ICC (%)	*P* value	Pathway
LRP1B	10.5	22.4	0.03	None
BRAF	11.1	0	0.01	RTK.RAS
EPCAM	0	4.0	0.01	None
GNA13	0	4.0	0.01	None
MYCL	0	4.0	0.01	None
PARP2	0	4.0	0.01	DDR
YES1	0	4.0	0.01	None
STK24	0.7	6.1	0.01	None
TSC1	2.0	8.1	0.04	PI3K

**Table 3:** BRAF annotation.

Variation type	Mutation effect	Oncogenic	Tumor	Drug
N581S	Gain-of-function	Oncogene	None	None
V600 E K601Q	Gain-of-function	Oncogene	None	None
D594 G	Gain-of-function	Oncogene	None	None
R260 C	None	None	None	None
K601 N	Gain-of-function	Oncogene	None	None
V600 E	Gain-of-function	Oncogene	Nonsmall cell lung cancer	Dabrafenib + trametinib
			Anaplastic	Vemurafenib
			Thyroid cancer melanoma	Dabrafenib
			Colorectal cancer	Vemurafenib + cobimetinib
			Hairy cell Leukemia	Trametinib
				Encorafenib + binimetinib
				Encorafenib + binimetinib + cetuximab
				Panitumumab + dabrafenib + trametinib
L597Q	Gain-of-function	Oncogene	None	None
V600 G	Gain-of-function	Oncogene	None	None
D594 N	Gain-of-function	Oncogene	None	None
Gene rearrangement	None	None	None	None
Splice sites change	None	None	None	None
G466 A	Gain-of-function	Oncogene	None	None

**Table 4:** Immune cell type of mutated gene in primary ICC and metastatic ICC patient.

Source	Type	Gene
Primary	B cells memory	CD1C; CD79 B; CD79 A; BLK; CD22; CD79 B; CD22; CD79 A; BLK; CD79 B; and CD22
	B cells naive	BRAF; BRAF; BRAF; BRAF; BRAF; BRAF; BRAF; BRAF; BRAF; BRAF; BRAF; BRAF; BRAF; BRAF; BRAF; BRAF; and BRAF
	Dendritic cells activated	BIRC3; MAP3K13; CD1E; MAP3K13; MAP3K13; MAP3K13; NR4A3; BIRC3; and MAP3K13
	Dendritic cells resting	CD1A and CD1A
	Macrophages M1	SOCS1
	Mast cells activated	NTRK1; MYB; and NTRK1
	Monocytes	HCK; CD1D; and HCK
	Neutrophils	CEACAM3; CEACAM3; and CEACAM3
	NK cells activated	CCND2; CDK6; CCND2; CDK6; and CDK6
	Plasma cells	PAX7
	T cells CD4 memory resting	ITK; IL7R; and ITK
	T cells follicular helper	TSHR and PDCD1
	T cells regulatory (Tregs)	CD70
Metastasis	B cells memory	CD79 A
	Dendritic cells activated	MAP3K13
	Mast cells activated	MYB and NTRK1
	Monocytes	HCK
	T cells CD4 memory resting	IL7R

## Data Availability

The data used to support this study are included within the article and in its supplementary files.

## Supplementary Materials

Supplementary Materials Figure S1: the ratio of patients harbored pathway gene mutation in primary ICC and metastasis ICC. *X*-axis descripted 11 pathway names, and *Y*-axis indicated that the ratio of patients with pathway gene mutation accounted for overall patients.
