# Supplementary material for: Somatic Mutation Profiling of Intrahepatic Cholangiocarcinoma: Comparison between Primary and Metastasis Tumor Tissues
Source: J Oncol. 2020 Sep 17;2020:5675020. doi: 10.1155/2020/5675020 (PMC7519439; doi:10.1155/2020/5675020)
Supplement: Supplementary Materials — Figure S1: the ratio of patients harbored pathway gene mutation in primary ICC and metastasis ICC. X-axis descripted 11 pathway names, and Y-axis indicated that the ratio of patients with pathway gene mutation accounted for overall patients. [file 5675020.f1.docx]

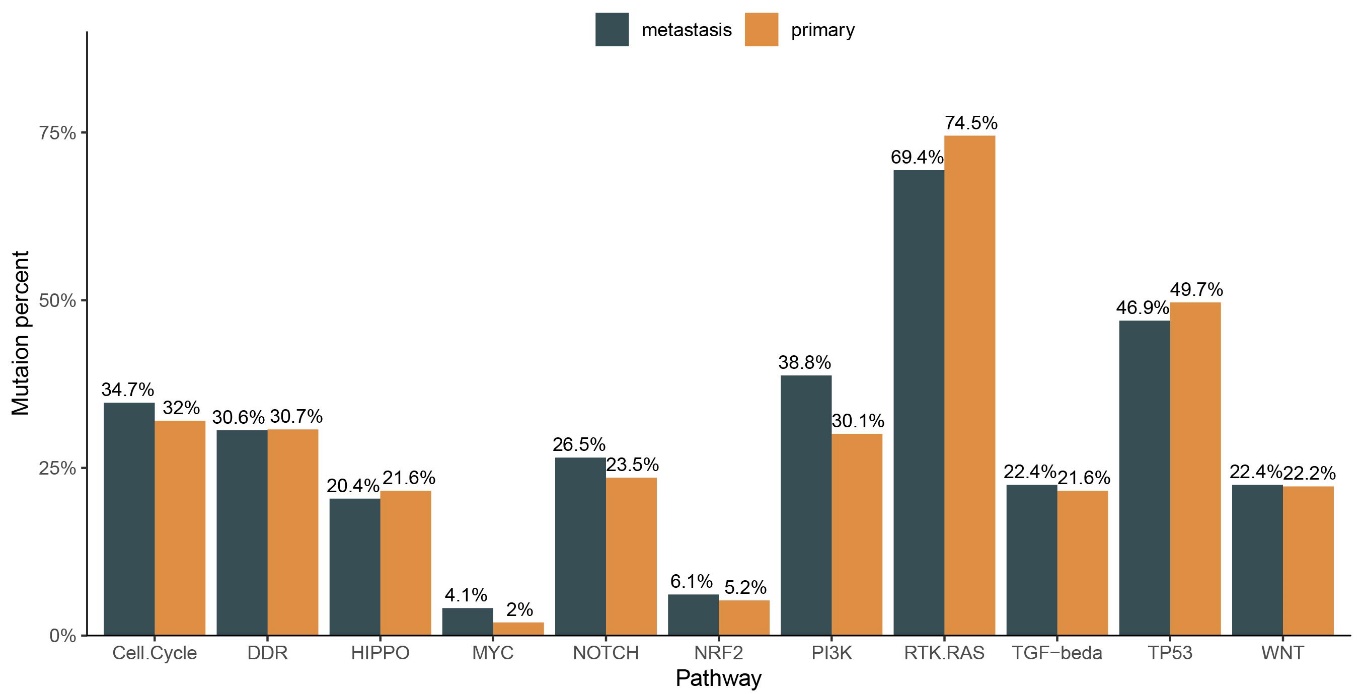
**Figure.S1 This figure showed the ratio of patients harbored pathway gene mutation in primary ICC and metastasis ICC. X-axis descripted 11 pathway names, and Y-axis indicated that the ratio of patients with pathway gene mutation accounted for overall patients.**
